# Deep Learning Hybrid Techniques for Brain Tumor Segmentation

**DOI:** 10.3390/s22218201

**Published:** 2022-10-26

**Authors:** Khushboo Munir, Fabrizio Frezza, Antonello Rizzi

**Affiliations:** Department of Information Engineering, Electronics and Telecommunications (DIET), Sapienza University of Rome, Via Eudossiana 18, 00184 Rome, Italy

**Keywords:** brain tumors, clinical diagnosis, convolutional neural networks, artificial intelligence, deep learning

## Abstract

Medical images play an important role in medical diagnosis and treatment. Oncologists analyze images to determine the different characteristics of deadly diseases, plan the therapy, and observe the evolution of the disease. The objective of this paper is to propose a method for the detection of brain tumors. Brain tumors are identified from Magnetic Resonance (MR) images by performing suitable segmentation procedures. The latest technical literature concerning radiographic images of the brain shows that deep learning methods can be implemented to extract specific features of brain tumors, aiding clinical diagnosis. For this reason, most data scientists and AI researchers work on Machine Learning methods for designing automatic screening procedures. Indeed, an automated method would result in quicker segmentation findings, providing a robust output with respect to possible differences in data sources, mostly due to different procedures in data recording and storing, resulting in a more consistent identification of brain tumors. To improve the performance of the segmentation procedure, new architectures are proposed and tested in this paper. We propose deep neural networks for the detection of brain tumors, trained on the MRI scans of patients’ brains. The proposed architectures are based on convolutional neural networks and inception modules for brain tumor segmentation. A comparison of these proposed architectures with the baseline reference ones shows very interesting results. MI-Unet showed a performance increase in comparison to baseline Unet architecture by 7.5% in dice score, 23.91% insensitivity, and 7.09% in specificity. Depth-wise separable MI-Unet showed a performance increase by 10.83% in dice score, 2.97% in sensitivity, and 12.72% in specificity as compared to the baseline Unet architecture. Hybrid Unet architecture achieved performance improvement of 9.71% in dice score, 3.56% in sensitivity, and 12.6% in specificity. Whereas the depth-wise separable hybrid Unet architecture outperformed the baseline architecture by 15.45% in dice score, 20.56% in sensitivity, and 12.22% in specificity.

## 1. Introduction

The emergence of deep learning (DL) has brought a new age of data science study and development [1]. Within a relatively short period of time, DL has had an impact on every aspect of life. The greatest immediate impact is felt in image processing [2], robotics [3], self-driving vehicles [4], natural language processing [5], computer games [6], and many other fields. The excellent performance-to-cost ratio, along with the widespread availability of computer technology such as graphics processing units (GPUs) and multi-core processor chips, has made DL extremely popular among data scientists [7]. The cornerstone of DL is the formalization of the concept that all brain functions are generated from neural activity in the brain [8]. The McCulloch-Pitts neuron model is a groundbreaking investigation into the operation of neural networks that led to the creation of numerous additional neural models of the brain, i.e., feedback neural networks [9], feed-forward neural networks [10], perceptrons [11], etc. While previous networks were either single layer (input-and-output) or featured a single hidden layer (input-hidden-outputs), the DL paradigm takes advantage of adding depth to the network by utilizing multiple hidden layers. Learning can be supervised or unsupervised. In supervised learning, the algorithm is taught by a human observer using training data and associated with the ground truth (GT) desired output and the system learns to recognize complicated patterns at the end of training. Unsupervised learning is the process through which an algorithm learns to recognize complicated patterns and processes without the assistance of a human observer. There are several DL adaptations for medical imaging. Deep belief network (DBN) is a deep learning (DL) adaption for unsupervised learning in which the top two layers work as associative memory [12,13]. DBN has found major uses in the creation and recognition of images [14,15], video surviellance [16], and motion capture data [17].Based on its intended segmentation outcome and accuracy, the active contour model (ACM) has become a prominent technique in image segmentation [18]. A stable and robust segmentation method based on pre-fitting energy was developed by Ge et al. [19]. Autoencoder is a deep learning-based network that is used for unsupervised learning [20]. An autoencoder’s input and output layers are composed of the same number of nodes, with one or more hidden layers linking them. It is done particularly to train the hidden nodes to encode the input data in a certain form so that it may be regenerated from that representation. As a result, instead of using traditional GT, input data is used to train the autoencoder. The convolutional neural network (CNN) is a form of deep learning (DL) that is employed especially in computer vision [21]. It is inspired by the way the biological visual system works. CNNs, like the animal visual cortex, take advantage of spatially-local correlation by imposing a local connection pattern between neurons in neighboring layers. There are several CNN models available, including GoogleNet [22], AlexNet [21], and LeNet [23] etc. It has been observed that as the depth of a DL-based system increases, its performance stagnates and then rapidly deteriorates. Deep Residual Networks (DRNs) allow for increased depth without sacrificing performance [24]. Scan images of infected/abnormal areas are often obtained in medical imaging utilizing computed tomography (CT) [25], magnetic resonance imaging (MRI) [26] and ultrasound (US) [27]. In most cases, professional physicians are responsible for identifying diseased tissues or any abnormalities. The development of computer vision and machine learning (ML) has produced a new generation of technologies in disease computer-assisted diagnostics (CAD) (Table 1). Image segmentation becomes complicated by intensity inhomogeneity, slow segmentation speed, and narrow area of application which can be corrected by an additive bias correction (ABC) model [28]. In [29] authors created an active deformable model for cervical tumor identification in 2008. Suri and his colleagues developed a feature-based recognition and edge-based segmentation method for measuring carotid intima-media thickness (cIMT) in 2011 [30]. In 2014, the same group developed an ML-based method for ovarian tissue characterisation [31].

During the same year, an attempt was undertaken to create a CAD system for detecting Hashimoto thyroiditis in US pictures from a Polish population [46]. Suri and his colleagues created a method for semi-automated segmentation of carotid artery wall thickness in MRI in 2014 [47]. The selection of a specific set of feature extraction methods is part of the ML characterization process. Selected features are integrated, in various ways, by ML-based algorithms for successful characterization. An open loop feature extraction procedure usually yields poor results. The introduction of DL in medical imaging has reduced the necessity for feature extraction techniques, as DL systems create features internally, avoiding the ineffective feature extraction stage. Deformable models are commonly employed in segmentation to estimate the shape of an infected/abnormal area in a medical image [48]. However, the inclusion of noise or missing data in a picture reduces the accuracy of deformable models, resulting in a poor border shape. DL uses pixel-to-pixel characterization to determine the form of an infected/abnormal shape in an image. This enables the DL to give an accurate form delineation. For 3D segmentation (34), in ML, a 3D atlas feature vector is generated from each voxel (3D picture unit), coupled with probability maps, and then training/testing is performed to define the inferred shape [49]. Such feature vector estimation is job dependent and may not be accurate for various types of 3D datasets. Internal feature extraction is performed in DL to approximate the position of the desired shape. As a result, DL provides a generic technique for segmenting 3D images that may also be expanded to accommodate 4D data such as video. Unlike ML, which updates weights concurrently, DL weights are changed layer by layer during training. Weights are updated layer by layer, which aids in the training of DL systems.

Machine learning, particularly deep learning, has proliferated in the diagnostic imaging industry over the past decade [50]. Deep learning algorithms, also known as deep neural networks, are constructed by stacking huge numbers of discrete artificial neurons, each of which performs elementary mathematical operations such as multiplication, summation, and thresholding. One of the fundamental factors behind the success of these new deep neural networks is the concept of representation learning, which is the process of automatically learning valuable characteristics from data as opposed to manual selection by experienced staff [1]. A convolutional neural network (CNN) is specifically intended to extract characteristics from two-dimensional grid data, such as images, using a sequence of learned filters and non-linear activation functions. This set of characteristics may subsequently be utilized to accomplish different downstream tasks such as image classification, object recognition, and semantic or instance segmentation [1]. Lately, U-Net [51], an end-to-end fully convolutional network (FCN) [52], was published for semantic segmentation of various structures in medical images. The U-Net design is composed of a contracting path that collects high-resolution, contextual data while downsampling at each layer, and an expanding path that boosts output resolution by upsampling at each layer [51]. Via skip connections, the features from the contracting path are joined with those from the expanding path, ensuring that the retrieved contextual characteristics are localised [53]. Originally designed for cell tracking, the U-Net model has lately been extended to additional medical segmentation applications such as brain vascular segmentation [54], brain tumor segmentation, and retinal segmentation [55]. In the medical picture segmentation literature, many multi-path architectures have been developed to retrieve features from provided data at different levels [37,56,57]. Inception modules have also achieved the notion of extracting and aggregating characteristics at multiple sizes [23]. Plain feature extraction techniques, however, differ from that of multi-path systems [37,56,57]. In this paper, we provide an end-to-end brain tumor segmentation system that combines a modified U-Net architecture with Inception modules to achieve multi-scale feature extraction. Furthermore, we assess the impact of training different models to directly segment glioma sub-regions rather than intra-tumoral features. All learning procedures were combined in a new loss function based on the Dice Similarity Coefficient (DSC). The suggested scheme is a fusion of CNN and U-net architecture. We propose four architectures and discuss their performance comparison. The first one is a recurrent-inception U-net network, the second is a recurrent-inception depth-wise separable U-net, the third architecture is a hybrid recurrent-inception U-net, and the fourth one is depth-wise separable hybrid-recurrent-inception U-net. Each one will be explained further in the paper. It is preferable to eliminate class imbalance using ROI detection for accurate segmentation. Using a CNN design, slices with tumor and no-tumor are categorized in the first stage. Following that, these slices containing tumors are sent to the network for pixel-by-pixel classification. The FLAIR and T2 MRI modalities are used by the classification network to highlight whole tumor areas, whereas the segmentation network uses all four modalities (i.e., T1, T2, T1c, and FLAIR). More details about these modalities are provided in Section 2.2.

## 2. Imaging Processing Techniques for Brain Tumors

Common challenges in cancer diagnosis are patient prognosis, grade estimation, surgery planning, and treatment response assessment. There are different forms of imaging techniques that are being used by doctors for different types of treatments. For brain cancer, two possible medical imaging techniques can be used and are known as functional and structural imaging [58,59]. Structural imaging is basically the measure of tumor location, brain structure, injuries, and other brain disorders. Conversely, the detection of lesions on a fine scale and metabolic changes, along with visualization of brain activities, is done in functional imaging. The metabolic changes which are reflected in the scans of the brain are used for activity visualization. Computed Tomography (CT) and Magnetic Resonance Imaging (MRI) is used for analyzing the human brain and assessing the presence and/or state of brain tumors [60,61,62].

### 2.1. Computed Tomography Imaging

In a CT scan, a specific body part is subjected to an X-ray beam capturing from various angles a series of images. Using this information, 2D cross-sectional images are created which are further combined to form 3D images providing a better view of the target organ. In cases of blood clots, hemorrhages, and cancer the most recommended procedure includes CT scans. A drawback is the use of harmful X-rays that emit ions having the ability to put a potential effect on living tissues, increasing the cancer risk. According to [63] the potential risk from CT radiations is 100 times more than a standard X-ray diagnosis.

### 2.2. Magnetic Resonance Imaging

MRI has good contrast providing fine details of the spinal cord, brain, and vascular anatomy. Furthermore, because there is no radiation involved, this technique is safer than CT. Visualization of the brain’s anatomy using MRI has three basic planes: coronal, sagittal, and axial. The sequences of MRI which are most commonly being used are T1-weighted, T2-weighted, and FLuid Attenuated Inversion Recovery (FLAIR) [64].

**Figure 1:**
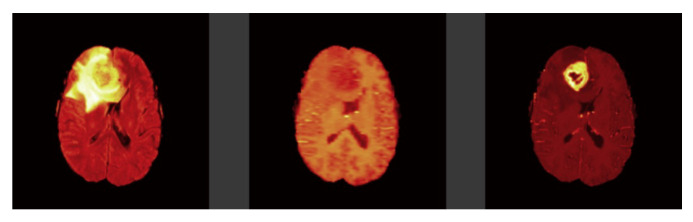
Brain tumor modalities. (Left image: FLAIR, Middle image: T1-weighted, and Right image: T1ce respectively).

**Figure 2:**
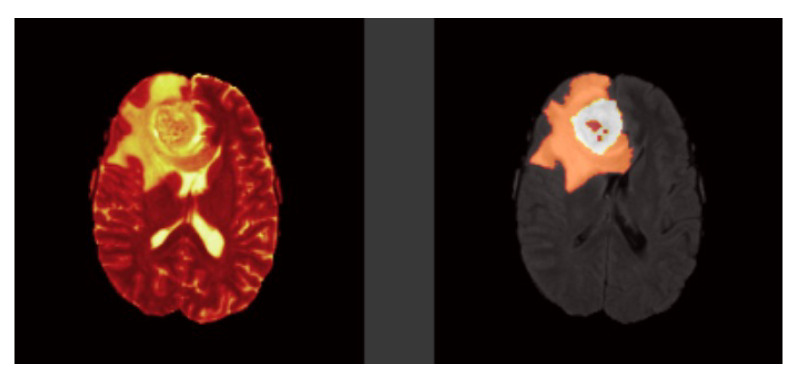
Brain tumor modalities. Left image: T2-weighted and Right image: Ground Truth.

Tl-weighted is basically used to distinguish between healthy and diseased tissues, and these scans offer a distinction between gray and white matter Figure 1. T2-weighted are well matched to brain disorders in which water accumulates within brain tissues due to the vulnerability of this modality to water content Figure 2. This modality determines the area of the edema, resulting in the generation of a bright signal on the image. Colorless fluid found in the spinal cord and brain known as cerebrospinal fluid (CSF) can be separated effectively using T1 and T2-weighted images. In T2-weighted images this CSF looks bright, and in T1-weighted images look dark. T1-weighted MRI with gadolinium contrast enhancement (T1-Gd) is the fourth type of MRI sequence. In this modality, an accumulated contrast agent, such as gadolinium ions, is used in the active cell area of the tumor tissues to produce a bright signal that facilitates the demarcation of the tumor boundaries. Since necrotic cells do not associate with contrast agents, they are segregated as a hypo-intense part of the center of the tumor, and this promotes the segmentation of the hypo-intense part of the active cell zone. FLAIR, with the exception of its acquisition protocol, is identical to T2-weighted images. Here the suppression of the water molecule is achieved, which in turn helps to discriminate between the edema and the CSF. FLAIR has the capacity to block water signals, and the hypertensive periventricular lesion is easily visible.

Brain tumors have a lower frequency as a neurosurgical illness than liver, esophageal, and breast tumors, but they cause a considerable increase in global mortality. Brain cancer is the worst kind of cancer in the world, killing a huge number of people, both adults and children. Due to the increased risk of death from this tumor, the development of diagnostic techniques has emerged as a key field of research. Early detection of a brain tumor improves a patient’s chances of survival. Tumor diagnosis aids doctors in deciding on the best treatment option, such as chemotherapy or surgery. Tumors are classified into four categories by the World Health Organization [65].

Benign tumors grow slowly and do not invade or spread. Gliomas and metastases account for approximately 80% of all malignant tumors [66]. Gliomas are divided into two types based on their aggressiveness: low-grade glioma (LGG) and high-grade glioma (HGG). MRI is a noninvasive imaging technique that may be used to diagnose and treat human tissues. MRI will give high-definition slices with exact information about the tumor and healthy areas [67,68]. Image segmentation is a critical topic of computer vision research. Long et al. [52] suggested a Fully Convolutional Network (FCN) to achieve this goal. It employs a deconvolutional layer to avoid recurrent storage issues and image size restrictions. Badrinarayanan et al. [69] developed a pixel-level segmentation network (SegNet) that considers spatial consistency and optimizes training using stochastic gradient descent, with an emphasis on memory consumption and computational efficiency. Zhang et al. [70] proposed a class-independent network (CANet). The complexity and objectivity of medical images impose significant constraints on image segmentation [70]. Examining these sections manually using the radiologist’s skills, depending on the pathologist’s experience, is a time-consuming procedure with a high mistake rate. This reduces the accuracy of the results, making them untrustworthy. Another important concern arises in the case of a misdiagnosis, which can cause substantial damage or reduce survival. Many medical diseases have been diagnosed using computer-aided methods, such as malignancies, COVID-19, and others [71,72]. As a result, employing computer-aided approaches to detect cancer is a valid alternative. This method’s most important phases are brain imaging slicing, feature extraction, and segmentation [73,74].

Segmenting medical images provides a solid pathological foundation while also assisting clinicians in making more accurate clinical diagnoses. Deep learning has recently been used in the field of brain tumor segmentation. In the following, a quick overview of the timeline development of segmentation algorithms in medical images is provided. Ronneberger et al. [51] proposed a U-Net network for the first time to segment biological images. U-Net is a network that shrinks together contextual information and integrates in-depth information with shallow rough information for completing end-to-end training using a cascade of encoder-decoder, which maximizes segmentation effectiveness.

Li et al. [75] created an H-DenseUNet to fuse the properties of CT slices of the liver. This network tackles the problems of a lack of context in 2-D space as well as the high cost of 3-D spatial calculation. Myronenko [76] proposed a three-dimensional method for MRI brain tumor segmentation using Encoder-Decoder architecture and a variable autoencoder to reconstruct an input image to improve regularisation to successfully resolve problems with the small number of labeled MRI images in the medical data system. Zhou et al. [77] presented a UNet++ network that used full-scale jump connections and deep supervision layering to accomplish performance optimization and parameter minimization by capturing in-depth distinct aspects of integration and superimposition. The research problem which we are addressing in this paper is the detection and segmentation of brain tumors with high efficiency. We propose novel architectures which will be compared to the conventional U-Net architecture. The U-Net architecture by Ronneberger et al. [51] will be used as a stepping stone for the architecture design. To improve the performance of segmentation, new architectures will be proposed and tested. A comparison of these proposed architectures with the baseline one will be carried out. In the end, the best performance will be compared to the state-of-the-art methods.

## 3. Dataset for Brain Cancer

**Figure 3:**
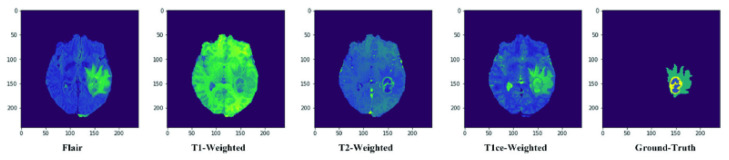
Dataset Samples from BraTS-2019.

The MICCAI BraTS training dataset from 2019 is used to train the segmentation model. It is composed of 335 patients and 4 distinct modalities (T1, T2, T1CE, and Flair). Some sample images are shown in Figure 3. NIfTI scans [78] have been obtained using a variety of clinical procedures, as well as a variety of scanners from 19 different institutions. All images were manually segmented by one to four raters, following the same annotation methodology, and the annotations were approved by expert neuroradiologists. Annotations’ labels concern the enhancing tumor (label 4), the peritumoral edema (label 2), the necrotic and non-enhancing tumor core (label 1), and everything else in the picture (label 0). Pre-processing included co-registration to the same anatomical template, interpolation to the same resolution (1 mm^3^), and skull-stripping [79,80,81]. Each patient is made up of 155 images termed slices of each modality. The data collection includes both low-grade glioma (LGG) and high-grade glioma (HGG) cases, with tumor classifications determined by specialists in the field. There are 76 LGG patients and 259 HGG patients in the data collection, for a total of 335 individuals.

### 3.1. Training, Testing, Validation

The 335 cases are shuffled to blend LGG and HGG patients. The shuffled data is then split into three subsets, with the training, validation, and test sets containing 235, 50, and 50 patients, respectively.

### 3.2. Imbalanced Data

Approximately 43% of the MR images in the training set include tumor tissue pixels. These magnetic resonance images will be utilized for training purposes. All images in this group containing tumor tissue may be reduced from the original size of 240×240 to 176×176 without losing important information. This cropping is prompted by a brute force method that checks whether the beginning and final pixels containing brain tissue are correctly positioned in both the horizontal and vertical directions of the brain picture for each sample. The 46% decrease in voxels results in no loss of information since all essential information is contained inside the brain pixels. Because of the reduced image size, this filtering, and cropping improves both class imbalance and computing time during training.

## 4. Software Environment

### 4.1. TensorFlow

TensorFlow [82], which was initially made available by the Google Brain team in 2015, is an open-source software library designed for formulating and executing machine learning algorithms. It was designed to be scalable, allowing computations to be done on many CPUs and GPUs for quicker calculations. TensorFlow is quick at performing matrix operations since it is written in C++; nevertheless, it can be accessed and controlled using other languages like Python and Java. Due to its accessibility, simplicity, and speed, it is now one of the most popular machine-learning libraries. There are additional libraries that can run on top of TensorFlow, one of which is Keras.

### 4.2. Keras

Keras [83] is a high-level neural network API developed in Python that can operate on top of TensorFlow. It enables quick and easy prototyping because of its user-friendliness, modularity, and extensibility. Keras is a TensorFlow-based framework that can operate on many CPUs and GPUs, providing scalability and performance.

### 4.3. 3D Slicer

3D Slicer is an open-source program that may be used for medical picture viewing and modification. The 3D slicer platform offers a wide range of applications for picture pre-and post-processing in a variety of medical areas. The user community maintains and develops the apps supplied by 3D Slicer. In this work, we used a 3D slicer for image preprocessing as well as 2D and 3D visualizations of the tumor and brain anatomy.

## 5. Image Data Preprocessing

Before a brain image can be studied further, many preprocessing procedures must be completed. Skull stripping is a term used to describe the process of separating the brain from extracranial or non-brain tissue in MR brain imaging investigations. Skull stripping removes information that is not useful when analyzing MR brain pictures for malignancies, useless from a machine learning standpoint. Other image processing methods such as co-registration to about the same anatomical template and interpolation to the same resolution should be used before skull-stripping of the brain areas [84].

### Spatial Resolution

The image sizes utilized in the network for training, validation, and testing must have the same characteristics in the overall machine learning processing stages. In the case of magnetic resonance images, different machines and clinical protocols produce images of varying sizes, necessitating the need to resample the images to the same size. Image resizing is a scaling process that falls under image registration and is performed using interpolation methods. There are several interpolation methods, but they all seek to add information by monitoring surrounding data to make an informed approximation. When comparing the gray-value errors of the 84 filters assessed in a publication on medical picture interpolation, the linear interpolation performs best for 2 grid points and the cubic convolution filter for 4 grid points. Grey-value errors are reduced by 28% to 75% when using cubic convolution. For larger filters, employing Welch, Cosine, Lanczos, and Kaiser windowed sinc filters yielded even better results with a decrease of 44–95%; however, these techniques generate heavier calculations, increasing computing time.

## 6. Method and Experiment

### 6.1. Recurrent-Inception UNET

Convolutional neural networks are characterized by two structural parameters: depth and width. Width denotes the filter number at every layer, whereas depth represents the number of layers. There will be an exponential increase in parameters to be tuned if more layers are incorporated within the network. Too many parameters can result in the overfitting of the network, whereas deep networks are more likely to experience a problem of vanishing gradient. Google Net used the bottleneck layer of convolution 1×1, which can be channel-wise, for map pooling to reduce map numbers with their high characteristics quality while overcoming large space parameters. Inception modules feature several filter sizes that aid in learning various types of variations found in distinct images to enhance the handling of multiple object scales. Firstly, the features learned from a layer are delivered to distinct routes; secondly, each path using the appropriate filter size learns features; and lastly, concatenation of the features from all the paths is carried out and passed down to the next layer.

#### Inception

Inception modules enhance network scalability by capturing data at multiple levels. As we get deeper into convolutional networks, the spatial concentration of features decreases. Large-sized kernels are important in the early stages for capturing more global information, but small-sized kernels are preferable in the latter stages for capturing more local information. Across the network, different inception modules are employed based on the variable dimension of features, since bigger filter sizes are more beneficial for learning key aspects of images with high spatial sizes while having an averaging impact on images with small spatial sizes. At the beginning of encoder, Inc.Block consists of high ratio large kernels 5×5 and 7×7 in comparison with small kernel 3×3. Within deeper levels, Inc.Block consists of a small ratio of large kernel 5×5 and 7×7 in comparison with small kernel 3×3. Furthermore, to solve the deep model’s delayed convergence difficulty, a batch normalization layer is employed after every inception layer for normalizing features.

**Figure 4:**
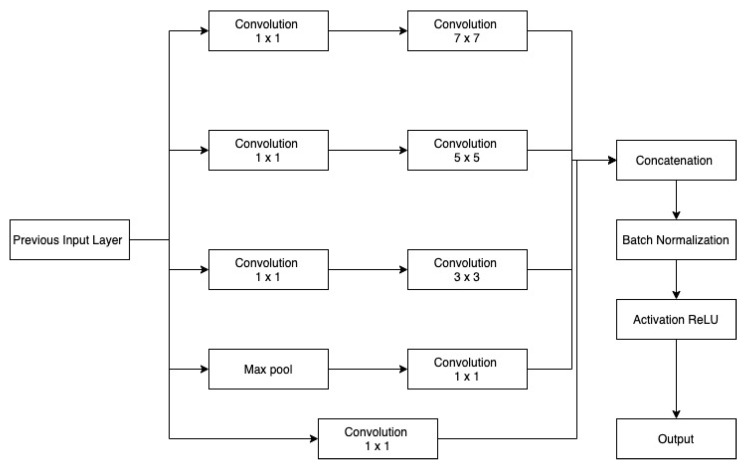
MI: Inception block 1.

**Figure 5:**
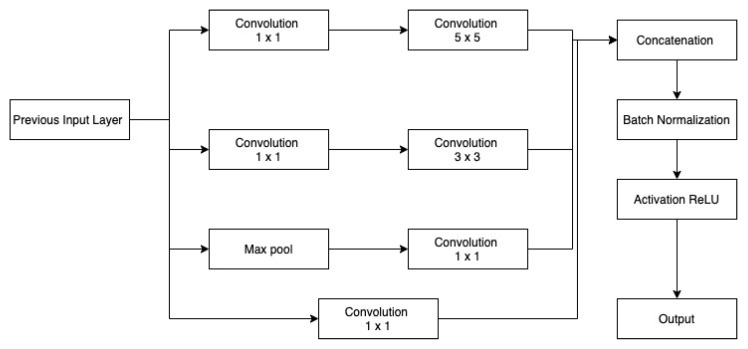
MI: Inception block 2.

**Figure 6:**
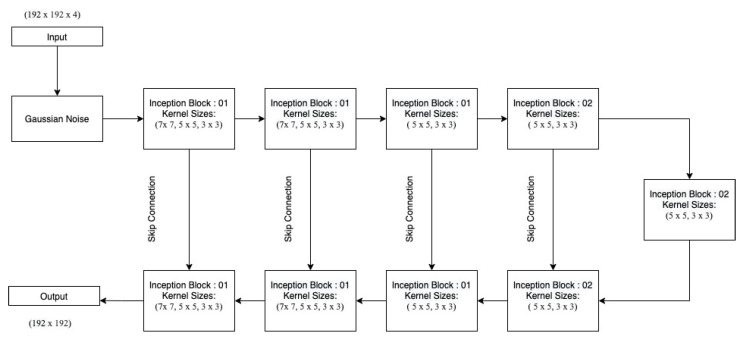
First proposed Architecture: Multi-Inception Model.

Figure 4 and Figure 5 depicts the first and second Inc.Block. In both blocks, different filter sizes are employed, and features from several branches are concatenated (Inception block). First Inc.Block consists of 5×5 and 7×7 sized filters, whereas in second Inc.Block learns small filters 3×3. The first proposed architecture is shown in Figure 6.

### 6.2. Recurrent-Inception UNET with DS-Convolution

#### Depthwise Seperable Convolution

**Figure 7:**
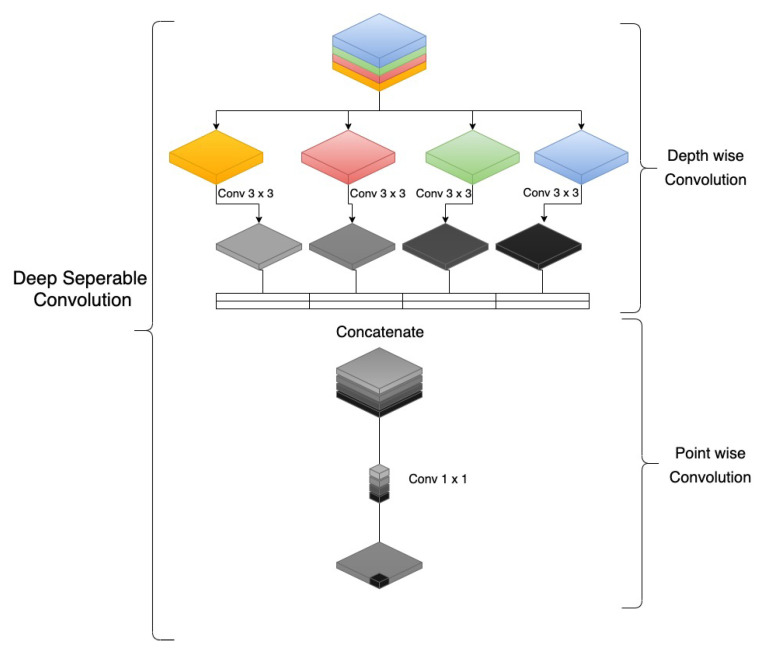
Depth-wise Separable Convolution.

Convolution is performed on all picture channels simultaneously by the standard convolution kernel. Each convolution kernel is associated with a feature map. A simultaneous learning of the deep and spatial convolutions occurs [85] (Figure 7).

$U_{h}$ represents the height and $U_{w}$ stands for the width of the convolution kernel. Let (*P*, *Q*, *C* ) be the input feature map, where *P* corresponds to width, *Q* is height, and *C* denotes the number of input channels. Consequently, the output feature size will be (*P*, *Q*, *D*) with *D* being the number of output channels. $Y_{1}$ is the standard convolution which is calculated as:

(1) $$Y_{1}=U_{w}\times U_{h}\times C\times D\times P^{2}$$

Depth-separable convolution includes point-wise and depth-wise convolution. The first one is in charge of filtering, while the second one is in charge of mapping output characteristics. For each channel, a deep convolution operates separately, and the 4 input channels in the 2D plane are combined with distinct kernels. Depth-wise convolution is calculated as:

(2) $$Y_{1}=U_{w}\times U_{h}\times C\times P^{2}$$

Point-wise convolution is calculated as $C\times D\times P^{2}$, whereas $Y_{2}$ denotes depth-wise separable convolution, calculated as the sum of depth-wise convolution and point-wise convolution,

(3) $$Y_{2}=U_{w}\times U_{h}\times C\times P^{2}+C\times D\times P^{2}$$

When only a single attribute is retrieved, the depth-wise separable convolution performs worse than the conventional convolution. However, as the network depth and number of extracted characteristics rise, depth-wise separable convolution can save a considerable amount of computation time [86]. Depthwise separable convolution can be calculated as follows:

(4) $$\frac{Y_{2}}{Y_{1}}=\frac{U_{w}\times U_{h}\times C\times P^{2}+C\times D\times P^{2}}{U_{w}\times U_{h}\times C\times D\times P^{2}}$$

(5) $$\frac{Y_{2}}{Y_{1}}=\frac{1}{D}\frac{1}{U_{w}\times U_{h}}$$

The proposed network employs the residual dense connection approach in the encoder-decoder network to address the issue of restricted numbers of information streams [87].

The residual dense block is a fundamental network unit, where the first convolution layer of the first encoder block is added to the first convolution layer of all forthcoming encoder blocks. It can improve the ability of feature propagation to better replicate the image. Between different network blocks, the full-scale skip connections are established [88]. As network depth rises, the amount of image features increases. Since so many convolutional layers would result in information redundancy, in our work implementation of fusion of local features is used before upsampling for extraction and fusion of effective features within every base unit. The U-Net framework must perform upsampling four times since having too many features between four connections would result in an unacceptably lengthy network training period. This work is based on the concept of a residual network. We send the context information from the first residual dense block to the following residual dense blocks and incorporate the global characteristics. As a result, SDCN-Net may acquire deep features in a hierarchical framework.

Shallow feature information may be extracted using depth-wise separable convolutional layers. The residual dense block network structure is made up of three major components: extracting shallow features, learning local adaptive features, and fusing global features. Global feature fusion and local features are combined for the reduction of dimensionality. The full-scale skipping connection used in the design of the SDCN-Net network module can improve network generalization and minimize network degradation. Among cascading operations, U-Net’s long skip connection and the short skip connection of the residual network are merged, thereby causing an effect on output results by the bottom layer [89].

We set the kernel size to 3×3, whereas the number of modal channels is set to 4. The computational performance benefit becomes increasingly apparent as the number of channels rises.

**Figure 8:**
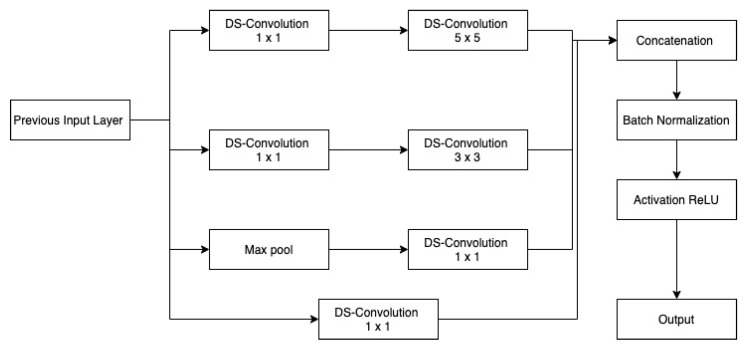
DS-inception block 1.

**Figure 9:**
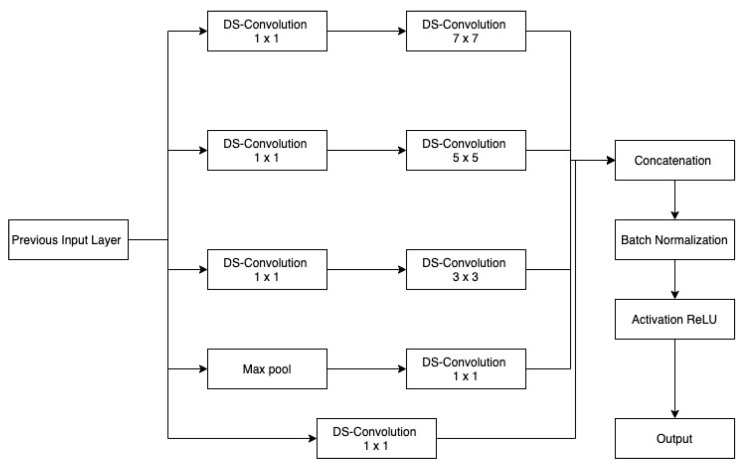
DS-inception block 2.

In the recurrent-inception network, the regular convolution operation is replaced by the depth-wise separable convolution operation. Inc.Blocks are identical to the ones given in the previous section (Figure 4 and Figure 5) with the only difference that here depthwise separable convolution is used as shown in the Figure 8 and Figure 9.

**Figure 10:**
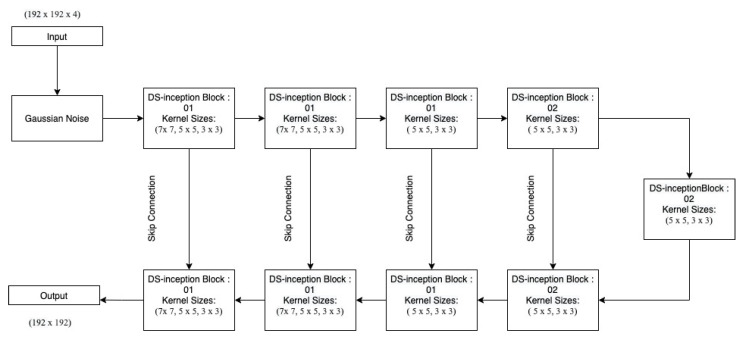
Second Architecture: Depthwise separable recurrent-inception Network.

The depth-wise separable recurrent-inception network is shown in Figure 10.

### 6.3. Hybrid Recurrent-Inception UNET

**Figure 11:**
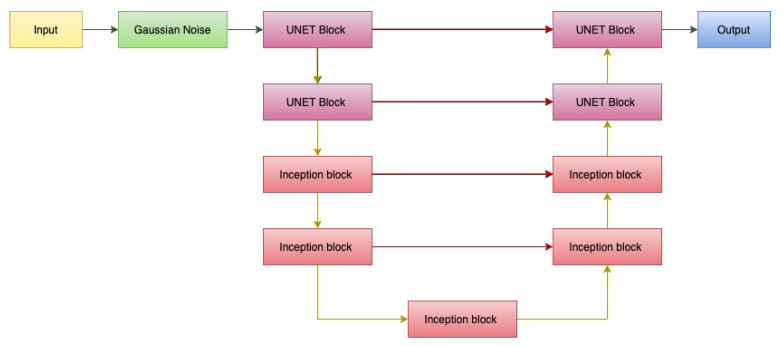
Third proposed architecture: Hybrid recurrent-inception UNET.

In the Hybrid recurrent-inception network, we combine the regular U-Net blocks, and the recurrent-inception blocks together to form a U-shaped architecture with skip connections. The design is shown in Figure 11.

#### DS-Hybrid Recurrent-Inception Unet

**Figure 12:**
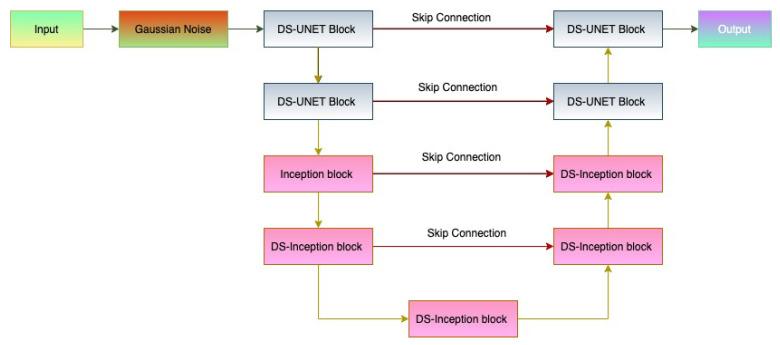
Fourth proposed architecture: Depth-wise separable Hybrid recurrent-inception UNET.

The depth-wise separable hybrid recurrent-inception U-net is shown in the Figure 12. It is identical to the hybrid recurrent-inception U-Net, except that the depth-wise separable convolution is used for depth-wise feature learning.

### 6.4. Experimental Setup

Each network was built using the backend of Tensorflow and the Keras framework. Furthermore, experiments are performed on a GPU-based system with 128 GB RAM and Nvidia K80 (12 GB VRAM).

The model was fed with cropped slices of size 240×240. Training of the classification network is carried out using optimizer Adam with learning rate $10^{\left(-4\right)}$, 200 epochs with 25 batch size. Class in Keras was used to initialize all of the convolutional layers in the segmentation UNET architecture.

Experiments were carried out utilizing a variety of CNN models with different numbers of convolution and dense layers. The CNN model which is optimized to achieve the best performance has nine layers in its design. Initially filter size of the convolution layers has size 3×3 with 32, 64, 128, 256, and 512 filters for the first five layers followed by fully connected layers. The activation function used in all layers is ReLU except the last one, where we used a sigmoid function. During training, we employed some data augmentation techniques such as horizontal flip, vertical flip, 10% zoom range, and 0.2 shear range.

All UNET settings are kept the same for Inception-UNET, except Inc.Block is inserted in every block. Features are acquired in the Inc.Block utilizing different scales of 3×3 and 7×7 kernels. To accomplish feature fusion, features were extracted from input by 7×7, 5×5, 3×3 and 1×1 convolutional layers, concatenated and batch-normalized to enhance convergence.

RI-UNET has various Inc.Blocks at various UNET levels depending upon spatial features concentration at every stage.

Because of the high spatial feature concentration at these stages, Inc.Block 1 is applied at the encoder’s first two stages and the later stages of the decoder. First Inc.Block consists of a higher number of large-sized filters than small ones. To accomplish feature fusion, features maps extracted from the input 1×1, 3×3, 5×5 and 7×7 are combined.

Due to the minimal spatial concentration of features at these levels, Inc.Block 2 is incorporated in the encoder’s latter stages and the first layers of the decoder. Here the small-sized filters are more in number than the large ones. To accomplish feature fusion, feature maps extracted from the input, 1×1, 3×3, and 5×5 convolutional layers are combined.

## 7. Results

This section summarizes the findings of the experiments presented in the methodology section. At first, the results of the training and validation dice coefficient, accuracy, and loss are presented for the selection of a learning rate for further simulations. Further, the training and validation performances of all four architectures presented in the previous section are presented.

The very first experiment is conceived to investigate the impact of varying learning speeds. The Equation (6) describes the normal distribution used for the initialization of weights, where μ=0 and σ=0.01.

(6) $$V_{ij}=N\left(\mu ,\sigma \right)$$

**Figure 13:**
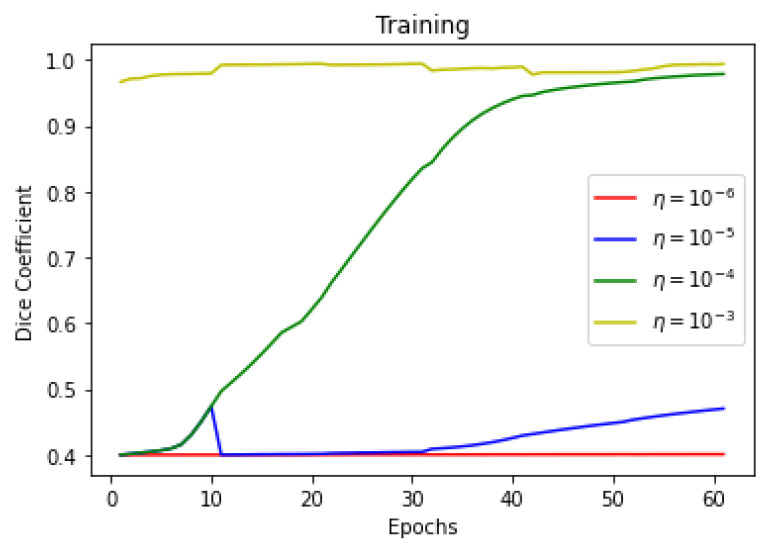
Training Dice Coefficient with Different Learning Rates.

**Figure 14:**
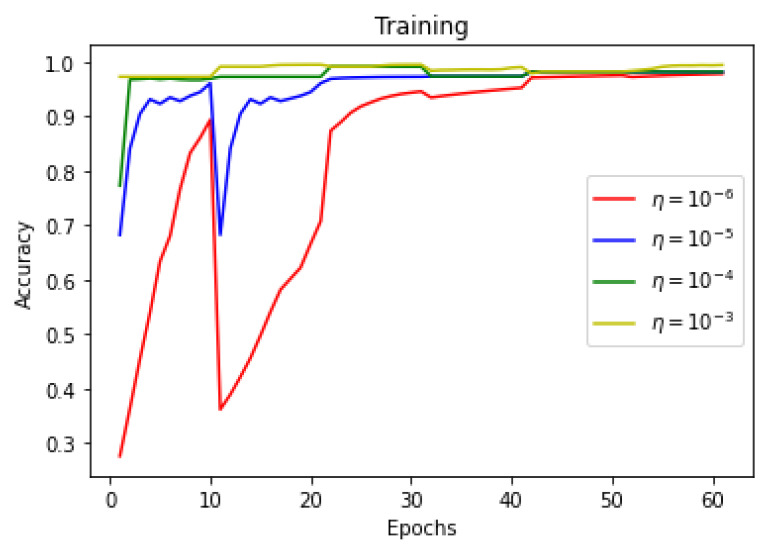
Training Accuracy with Different Learning Rates.

**Figure 15:**
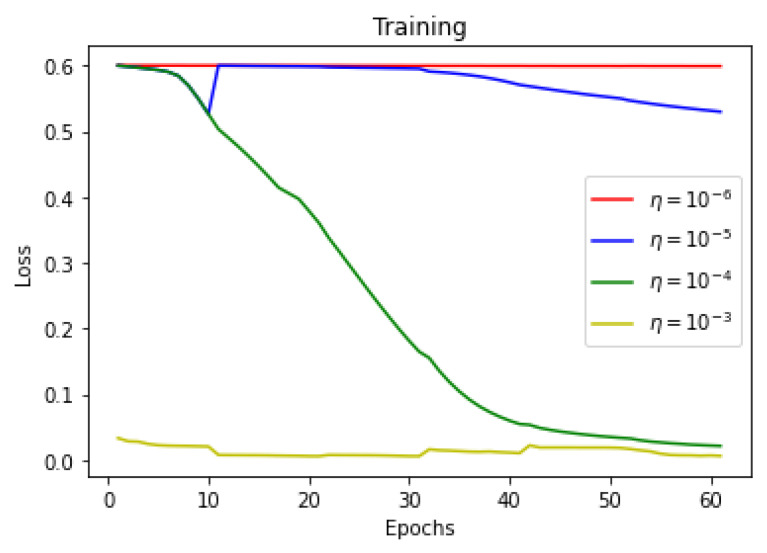
Training Loss with Different Learning Rates.

**Figure 16:**
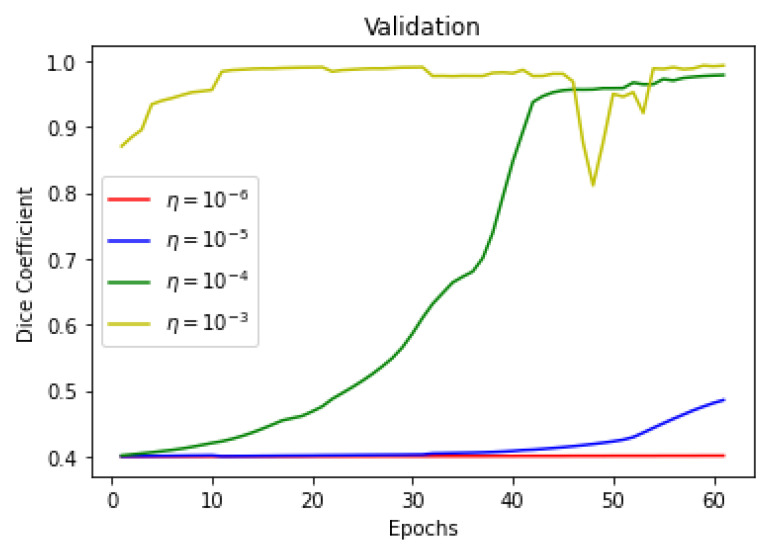
Validation Dice Coefficient with Different Learning Rates.

**Figure 17:**
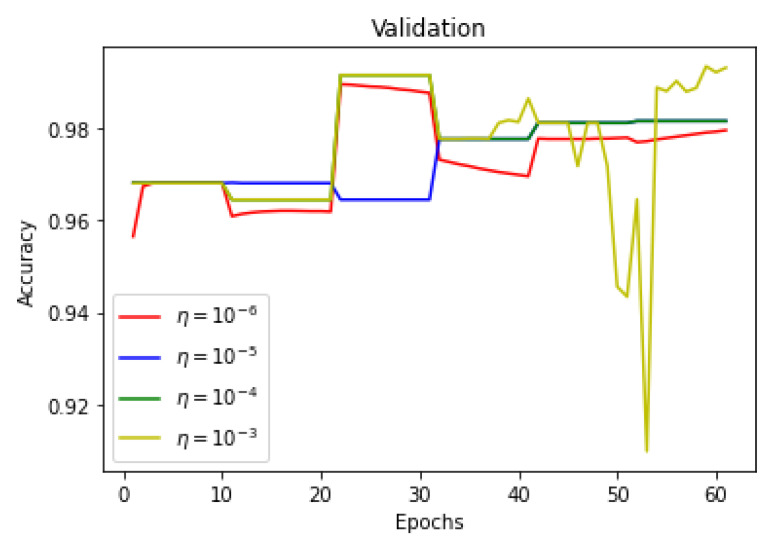
Validation Accuracy with Different Learning Rates.

**Figure 18:**
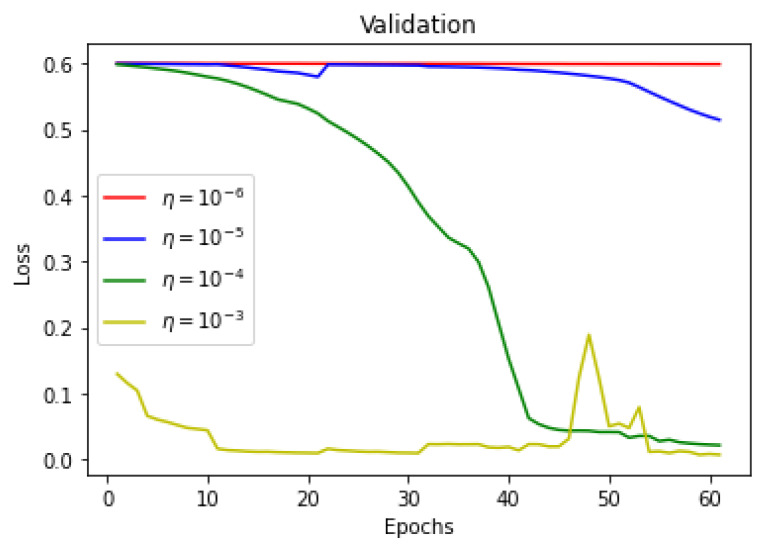
Validation Loss with Different Learning Rates.

As there is a limited amount of data, data augmentation is used to alter existing data in order to expand the available dataset. In Figure 13, Figure 14 and Figure 15 training dice coefficient, accuracy, and loss curves with respect to different learning rates are shown. In Figure 16, Figure 17 and Figure 18 validation dice coefficient, accuracy, and loss curves with respect to different learning rates are shown.

## 8. Comparison of Training, Validation, and Test Results

**Figure 19:**
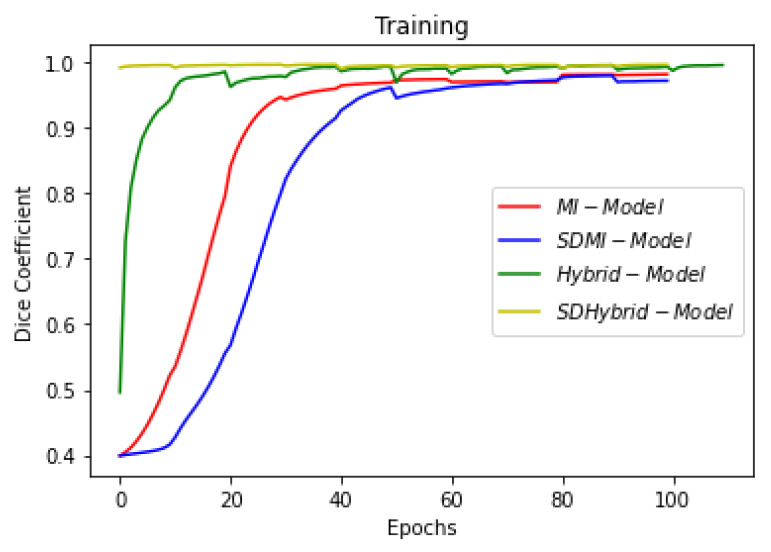
Comparison of Training Dice Coefficient.

**Figure 20:**
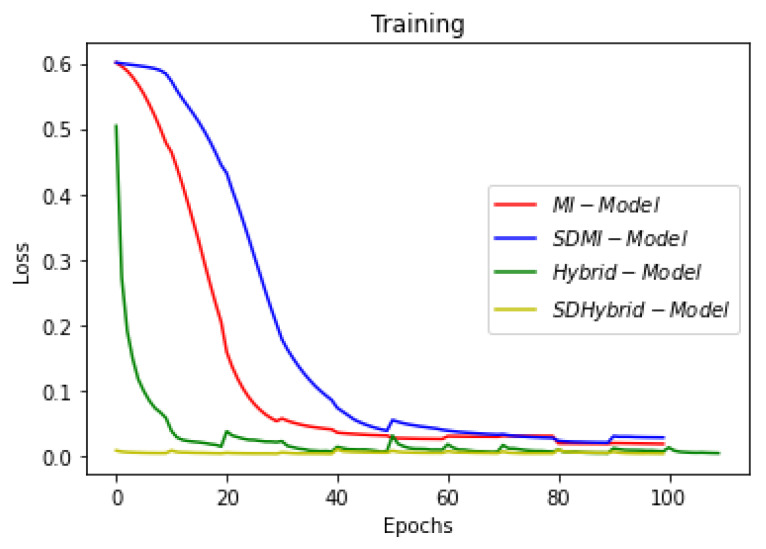
Comparison of Training Loss.

**Figure 21:**
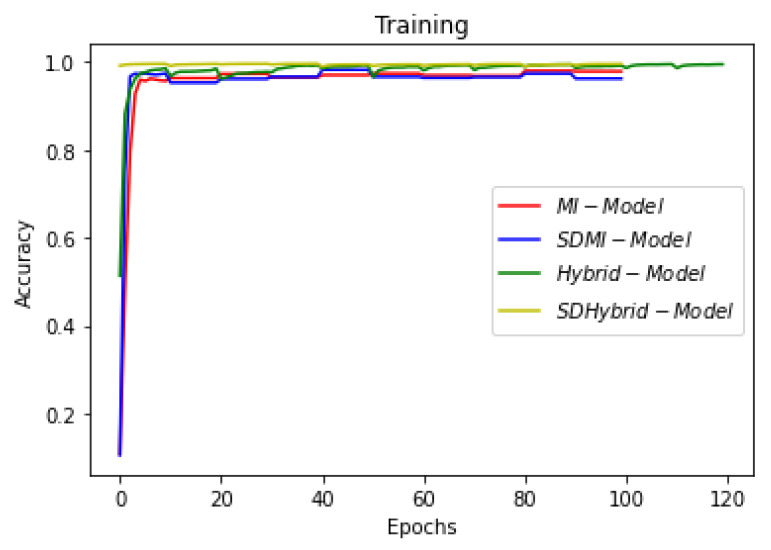
Comparison of Training Accuracy.

**Figure 22:**
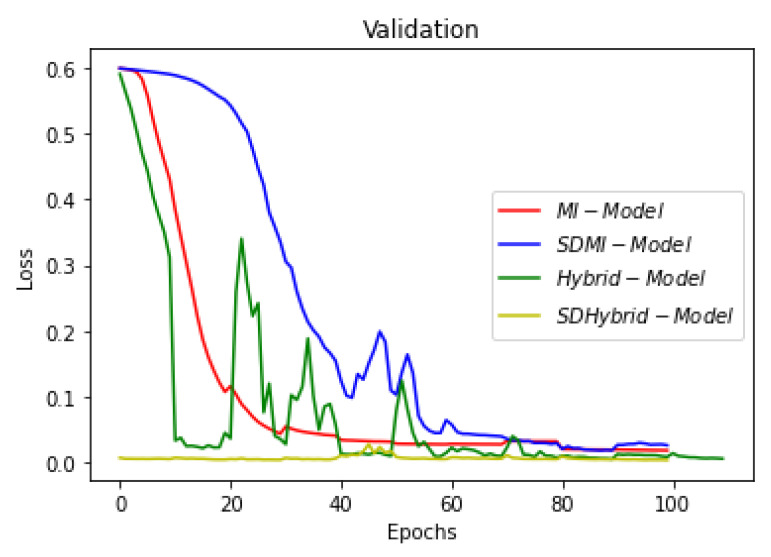
Comparison of Validation Loss.

**Figure 23:**
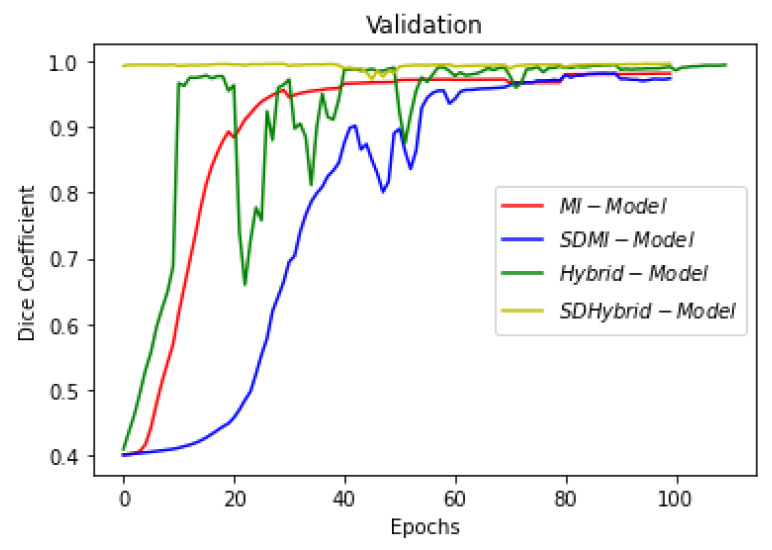
Comparison of Validation Dice Coefficient.

**Figure 24:**
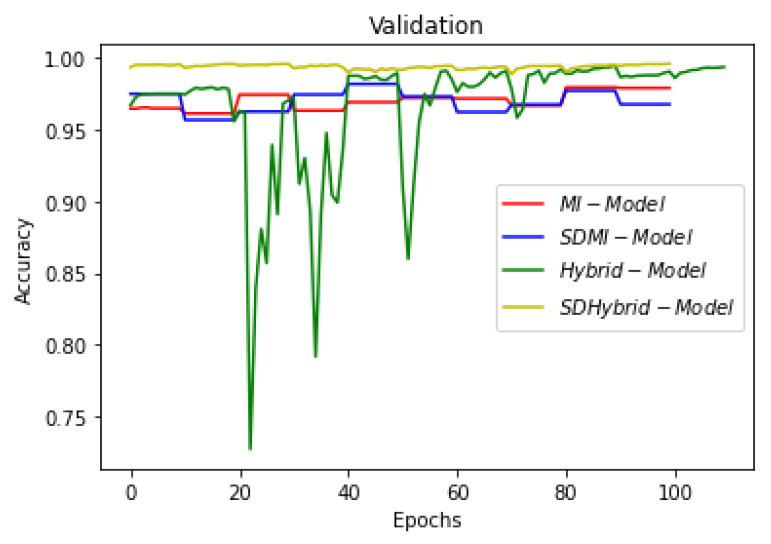
Comparison of Validation Accuracy.

**Figure 25:**
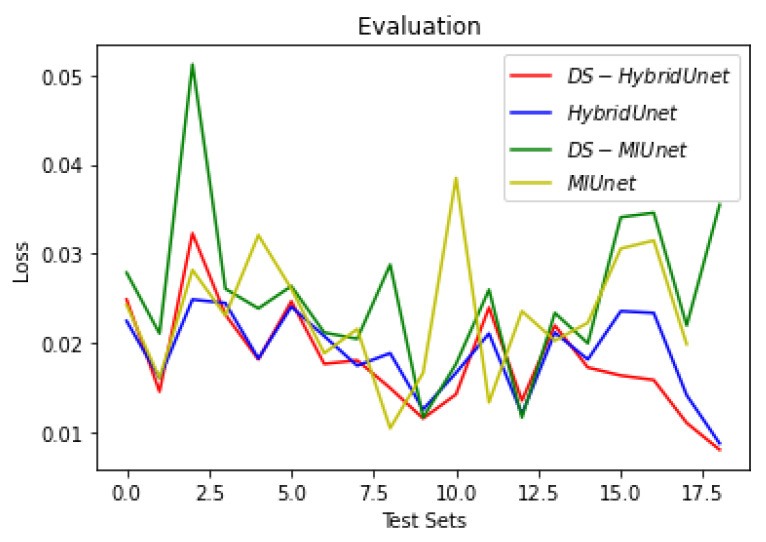
Comparison of Evaluation Loss.

**Figure 26:**
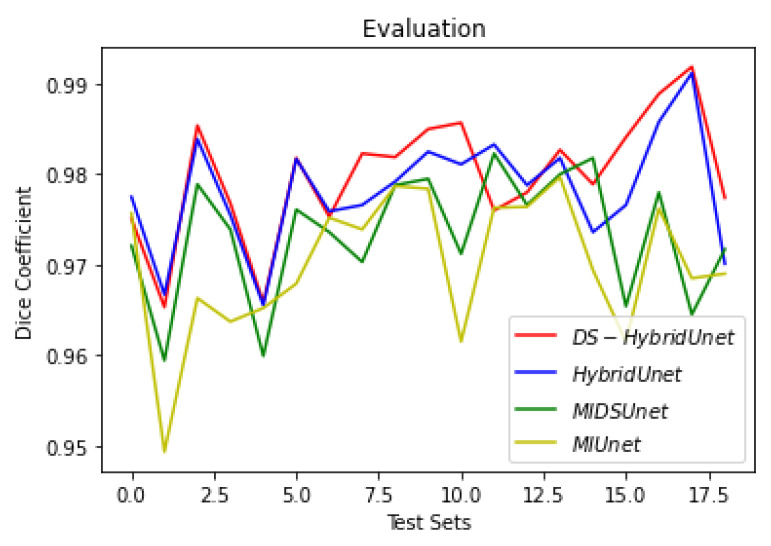
Comparison of Evaluation Dice Coefficient.

**Figure 27:**
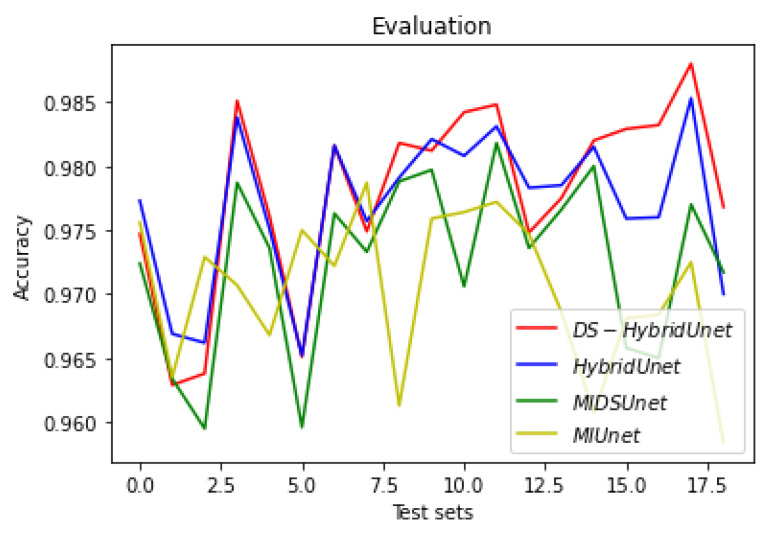
Comparison of Evaluation Accuracy.

Figure 19, Figure 20 and Figure 21 show the comparison of all four models when they are trained and validated using the same set of images. It can be seen that the depth-wise separable hybrid model performed better than the rest of the models. Similarly, Figure 22, Figure 23 and Figure 24 show the validation results. Figure 25 shows the loss of each model when tested on the test images. It can be seen that the MI-Unet and DS-MIUnet have almost similar loss curves, whereas the hybrid-Unet and DS-Hybrid Unet have low values of loss showing better performance than the rest of the models. Figure 26 and Figure 27 show the dice coefficient and accuracy for these models when evaluated on test images. It can be observed that Hybrid Unet and DS-Hybrid Unet outperformed the MI-Unet and DS-MIUnet with a remarkable increase in performance.

**Figure 28:**
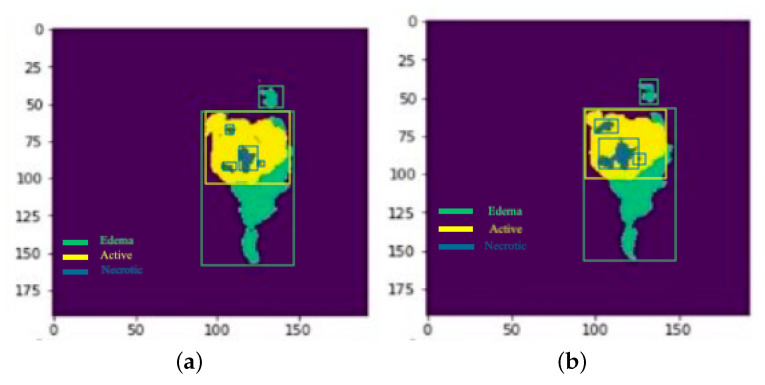
Results: Slice 1: (**a**) Actual result (**b**) Predicted result.

**Figure 29:**
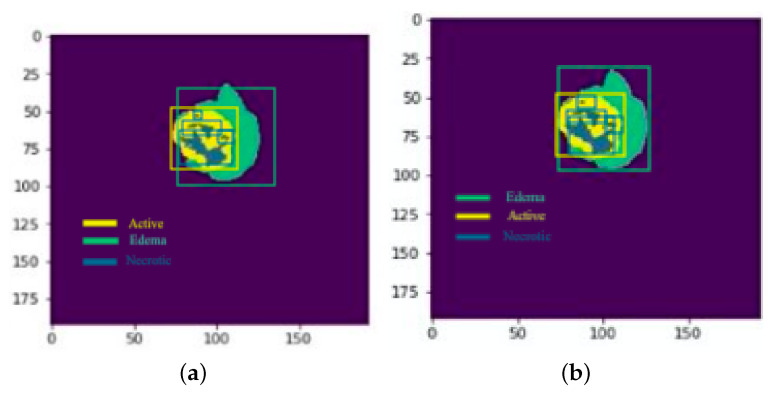
Results: Slice 2: (**a**) Actual result (**b**) Predicted result.

**Figure 30:**
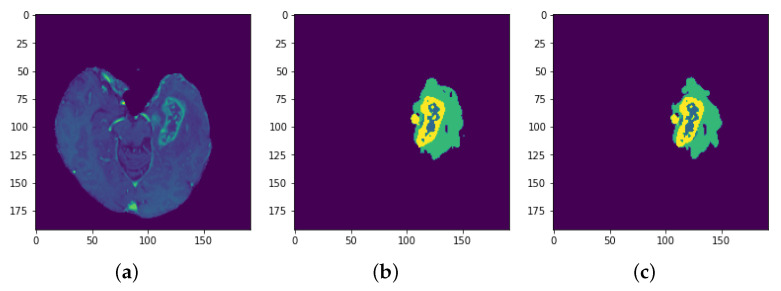
Results: Slice 3: (**a**) Slice (**b**) Predicted result (**c**) Actual result.

**Figure 31:**
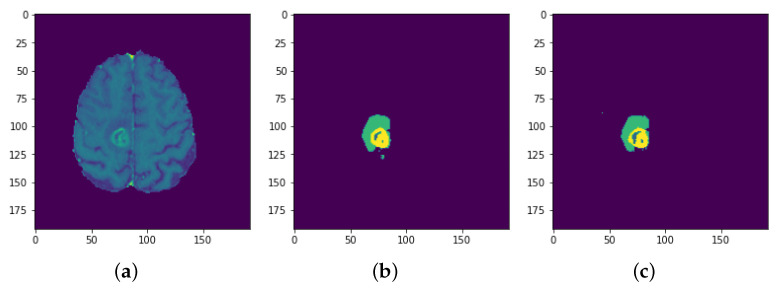
Results: Slice 4: (**a**) Slice (**b**) Predicted result (**c**) Actual result.

**Figure 32:**
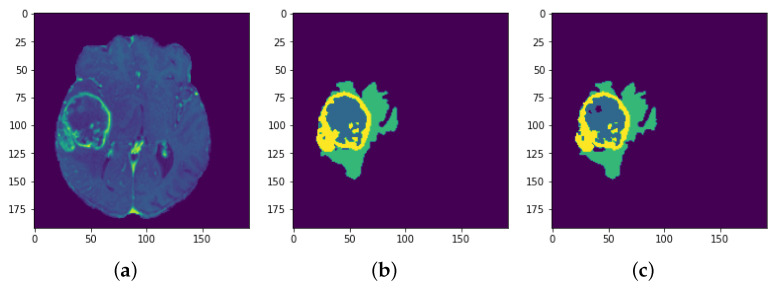
Results: Slice 5: (**a**) Slice (**b**) Predicted result (**c**) Actual result.

**Figure 33:**
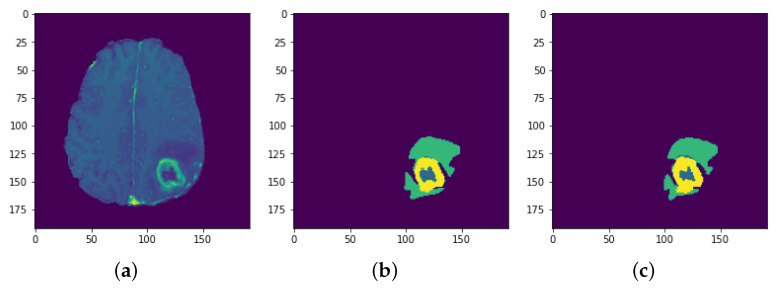
Results: Slice 6: (**a**) Slice (**b**) Predicted result (**c**) Actual result.

Each model is tested on the test images and the results are compared and are shown in Table 2. The difference in performance with each model is calculated and shown in the following tables. In Table 3 the baseline architecture is compared with the four proposed architectures. There has been a considerable performance improvement (the visual results are shown in Figure 28, Figure 29, Figure 30, Figure 31, Figure 32 and Figure 33). From Table 3 it can be seen that MI-Unet showed a performance increase in comparison to baseline Unet architecture by 7.5% in dice score, 23.91% insensitivity, and 7.09% in specificity. Depth-wise separable MI-Unet showed a performance increase by 10.83% in dice score, 2.97% in sensitivity, and 12.72% in specificity as compared to the baseline Unet architecture. Hybrid Unet architecture achieved performance improvement of 9.71% in dice score, 3.56% in sensitivity, and 12.6% in specificity. Whereas the depth-wise separable hybrid Unet architecture outperformed the baseline architecture by 15.45% in dice score, 20.56% in sensitivity, and 12.22% in specificity.

In Table 4 the proposed architectures are compared with MI-Unet architecture. It can be seen from the table that Depth-wise Separable MI-Unet architecture increased by 3.33% in dice coefficient, and specificity is increased by 5.63%. The hybrid model improved the dice coefficient up to 2.21% as compared to the MI-Unet and specificity is increased by 7.95%. A decrease in sensitivity can be seen in comparison with MI-Unet architecture.

Table 5 shows the results of a comparison of depth-wise separable MI-Unet architecture with the Hybrid model and Depth-wise separable Hybrid model. There is improvement in performance by 1.12% in dice coefficient score, 0.59% in sensitivity, whereas improvement by 4.62% in dice coefficient and 17.59% in sensitivity can be seen with depth-wise separable Unet, when compared with depth-wise separable MI-Unet architecture. A small decrease can be seen by 0.12% and 0.22% in sensitivity with respect to hybrid Unet and depth-wise separable Unet architectures, respectively.

Performance comparison of depth-wise separable hybrid U-net architecture with hybrid Unet one is shown in Table 6. Improvement in dice coefficient by 5.74% and sensitivity 17% can be highlighted, with a slight decrease in specificity by 0.38%.

Overall, it can be concluded that the SD-Hybrid model outperforms all other models presented in this work. At the end, we compared our results with state of the art methods.

To identify tumor pixels, a probabilistic approach that combines sparse representation and a Markov random field has been suggested in [90]. Random decision trees are trained on image characteristics in [91] to categorize voxels. The experimental findings obtained in this study are compared to state-of-the-art approaches in the benchmark brain tumor segmentation challenge [79] and methods presented in [79,90,91] as shown in the Table 7. The suggested model’s high specificity values indicate that it is effective in detecting the primary tumor area, avoiding false positives. It is evident that the proposed architectures performed better than the state of the art presented in Table 7.

## 9. Conclusions and Future Work

We set out to solve a difficult challenge concerning the segmentation of brain cancers from three-dimensional MRI scans. We studied the deep learning methods and decided to use a convolutional neural network for the development of suitable solutions for the said research problem. For brain tumor segmentation we created a new framework based on the well-known U-Net architecture and Inception modules. Our model resulted in a significant amount of gain in validation accuracy. We believe that the observed gain in validation accuracy is due to the use of several convolutional filters of varying sizes in each Inception module. During the learning process, these filters are capable of capturing and retaining contextual information at different scales. We further believe that the increased tumor segmentation accuracy is due to the new loss function based on the improved DSC. We assess our models using DSC in our proposed framework, and the learning goal or loss function (Dice loss function) used to train these algorithms is likewise based on DSC.

Extensive studies revealed that each element of the proposed framework achieves its intended purpose in identifying the complex patterns associated with tumor areas and contributes to better segmentation outcomes. In addition, the technique described in this paper might be improved further by using cascaded, ensembled, or other learning techniques. The main findings of this paper can be generalized and applied to a wide range of other biological image segmentation and classification problems, such as image registration, and disease quantification, as well as to other application fields.

## 10. Ethics

No animal or human beings were physically present/required in this study. No privacy of the patient was violated. Dataset was in the form of images. No live specimens were included.

## Figures and Tables

**Table 1:** Applications of deep learning (Segmentation (Seg.) and Classification (Classif.)).

Field	Data (Size)	Application (DL Scheme (Ref.))	Performance
Cardiovascular [32]	US (496)	left ventricle Seg. (DBN)	JD: 0.83, AV: 0.91, MAD: 0.95,
Cardiovascular [33]	MRI (45)	left ventricle Seg. (CNN)	AVP: 0.83 DM: 0.94 ± 0.02, APD: 1.81 ± 0.02 mm, Conformity: 0.86
Cardiovascular [34]	3D TEE (2891)	left ventricle Seg. fully Connected Network	Position Err: 1.47 mm Corner Err: 2.80 mm
Cardiovascular [35]	X-ray (44)	Vessel Seg. (CNN)	Acc: 93.5%
Cardiovascular [36]	US (56)	Plaque Classif. (CNN)	Acc: 0.75 ± 0.16
Neurology [37]	3D TBI (66)	Brain lesion Seg. (CNN)	DM: 0.59
Neurology [38]	MRI (339)	Brain lesion Seg. (CNN1 (HGG) + CNN2 (LGG))	DM complete: 0.88; DM core: 0.83; DM enhancing: 0.77
Mammography [39]	MITOS (50)	Mitosis Classif. CNN1 (11 layers) + CNN2 (13 layers)	Precision: 0.88; F1-score: 0.78
Mammography [40]	Dig. Mam. (219)	Breast tumor Classif. CNN + SVM	AUC: 0.86
Microscopy [41]	Mammalian cell lines (5)	Cellular Seg. CNN	JI (MN): 0.89; DM (MN): 0.95
Microscopy [42]	Breast cancer (340) Yeast (2480)	Cellular Seg. CNN + MIL	Breast cancer; Acc: 0.971; Yeast dataset; Acc: 0.963
Dermatology [43]	Dermatology (1279)	Melanoma Seg. FCN	Acc: 76%
Dermatology [44]	CT (30)	Liver tumor Seg. CNN	DM: 80.06%
Pulmonary [45]	CT (1010)	Lung Cancer Classif. CNN	CNN Sens: 73.3%

**Table 2:** Performance Comparison.

Architecture Names	Dice Coefficient	Sensitivity	Specificity
Baseline U-net	0.7230	0.6970	0.8720
Recurrent-inception U-net (MI-Unet)	0.7980	0.9361	0.9429
Depth-wise Separable MI-Unet	0.8313	0.7267	0.9992
Hybrid Model	0.8201	0.7326	0.9980
Depth-wise separable Hybrid model	0.8775	0.9026	0.9942

**Table 3:** Percentage Increase In Performance With Comparison to Baseline Architecture.

Architecture Names	Dice Coefficient	Sensitivity	Specificity
recurrent-inception U-net (MI-Unet)	7.5%	23.91%	7.09%
Depth-wise Separable MI-Unet	10.83%	2.97%	12.72%
Hybrid Model	9.71%	3.56%	12.6%
Depth-wise separable Hybrid model	15.45%	20.56%	12.22%

**Table 4:** Percentage Increase In Performance With Comparison to MI-Unet Architecture.

Architecture Names	Dice Coefficient	Sensitivity	Specificity
Depth-wise Separable MI-Unet	3.33%	20.94%	5.63%
Hybrid Model	2.21%	20.35%	5.51%
Depth-wise separable Hybrid model	7.95%	3.35%	5.13%

**Table 5:** Percentage Increase in Performance with respect to Depth-wise Separable MI-Unet Architecture. (“−” sign shows the decrease of specificity w.r.t. Depth-wise Separable MI-Unet Architecture).

Architecture Names	Dice Coefficient	Sensitivity	Specificity
Hybrid Model	1.12%	0.59%	−0.12%
Depth-wise separable Hybrid model	4.62%	17.59%	−0.22%

**Table 6:** Percentage Increase in Performance with respect to Hybrid U-net Architecture.

Architecture Names	Dice Coefficient	Sensitivity	Specificity
Depth-wise separable Hybrid model	5.74%	17%	−0.38%

**Table 7:** Performance Comparison with state-of-the-art methods.

Architecture Names	Dice Coefficient	Sensitivity	Specificity
SR [90]	0.7800	0.8500	-
RDF [91]	0.7900	0.8000	0.8200
Doyle [79]	0.7100	0.6600	0.8700
Festa [79]	0.7200	0.7700	0.7200
Baseline U-net	0.7230	0.6970	0.8720
Recurrent-inception U-net (MI-Unet)	0.7980	0.9361	0.9429
Depth-wise Separable MI-Unet	0.8313	0.7267	0.9992
Hybrid Model	0.8201	0.7326	0.9980
Depth-wise separable Hybrid model	0.8775	0.9026	0.9942

## Data Availability

The data was taken from MICCAI Public repository.
